# Supplementary material for: Saccharomyces boulardii (CNCM I-745) improves intestinal damage in sepsis by remodeling intestinal flora structure
Source: Front Cell Infect Microbiol. 2025 Jul 31;15:1602792. doi: 10.3389/fcimb.2025.1602792 (PMC12350329; doi:10.3389/fcimb.2025.1602792)
Supplement: Supplementary file 1 [file DataSheet1.zip › summary/summary/8_Advanced_analyse/2_PICRUSt2_result/KEGG/Group.DW4_vs_DW0/PICRUSt2_KEGG_level3_Group.DW4_vs_DW0_diff.pdf]

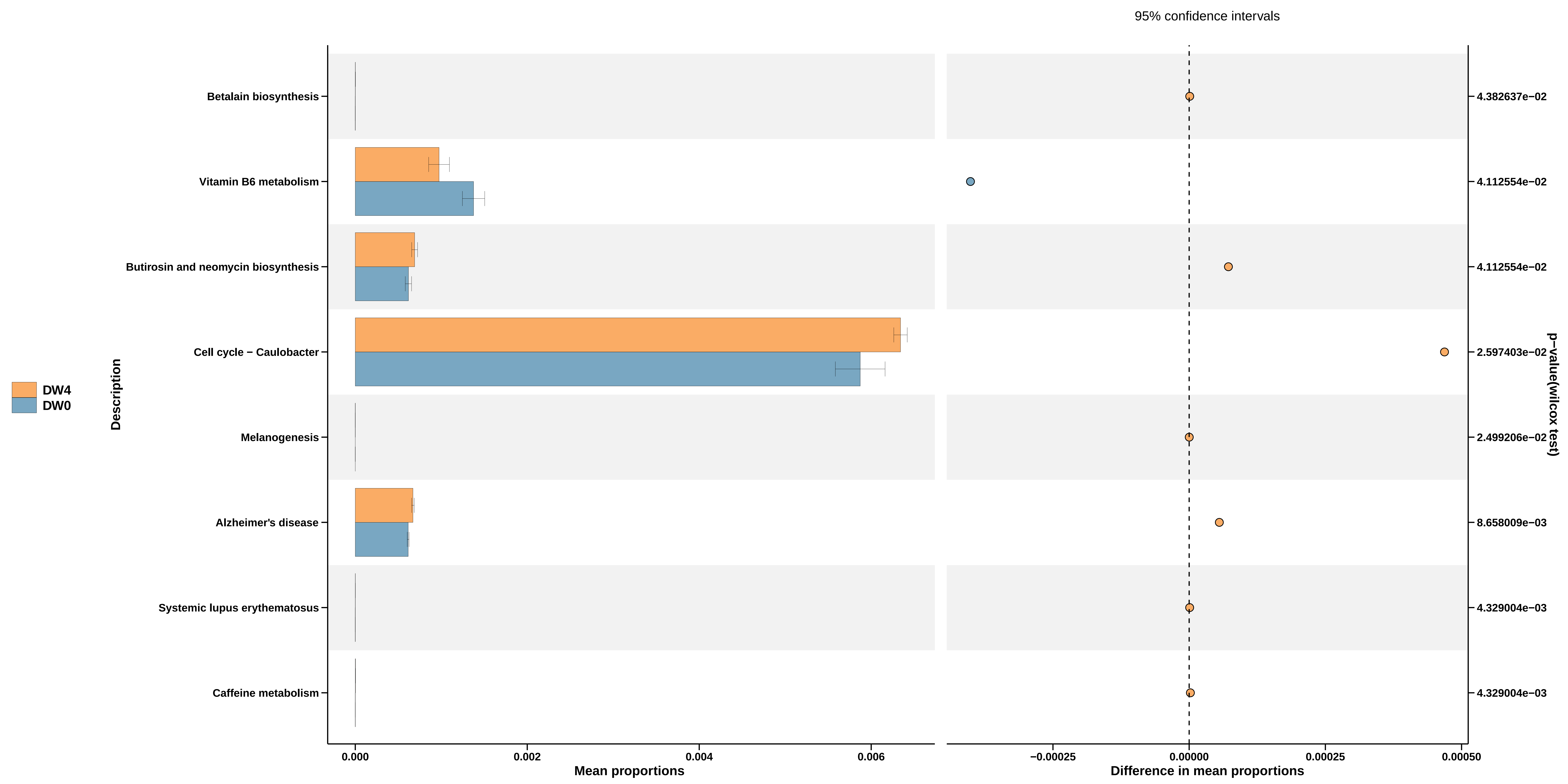

95% confidence intervals
Betalain biosynthesis
Vitamin B6 metabolism
Butirosin and neomycin biosynthesis
Cell cycle − Caulobacter
Melanogenesis
Alzheimer's disease
Systemic lupus erythematosus
Caffeine metabolism
4.382637e−02
4.112554e−02
4.112554e−02
2.597403e−02
2.499206e−02
8.658009e−03
4.329004e−03
4.329004e−03
DW4
DW0
Description
0.000
0.002
0.004
0.006
Mean proportions
−0.00025
0.00000
0.00025
0.00050
Difference in mean proportions
p−value(wilcox test)
